# The Deubiquitinating Enzyme *MrUbp14* Is Involved in Conidiation, Stress Response, and Pathogenicity in *Metarhizium robertsii*

**DOI:** 10.3389/ffunb.2022.896466

**Published:** 2022-05-16

**Authors:** Zhangxun Wang, Hua Chen, Hao Li, Hanyuan Chen, Bo Huang

**Affiliations:** ^1^Anhui Provincial Key Laboratory of Microbial Pest Control, Anhui Agricultural University, Hefei, China; ^2^Key Laboratory of Biology and Sustainable Management of Plant Diseases and Pests of Anhui Higher Education Institutes, School of Plant Protection, Anhui Agricultural University, Hefei, China

**Keywords:** *metarhizium*, deubiquitinase (DUB), Ubp14, virulence, protein ubiquitination

## Abstract

Protein ubiquitination, which is involved in various biological processes in eukaryotic cells, is a reversible modification of proteins. Deubiquitinases can maintain ubiquitin homeostasis by removing ubiquitin or modulating protein degradation *via* the ubiquitin-proteasome system (UPS). *Metarhizium robertsii*, an entomopathogenic fungus, has become a model fungus for investigating the interactions between insects and fungal pathogens. To explore the possible effects of the deubiquitination process on the development, stress response, and virulence of *M. robertsii*, disruption of *MrUbp14* (an ortholog of the yeast ubiquitin-specific protease gene, *Ubp14*) was performed. The results of this study showed that the deletion of *MrUbp14* led to accelerated conidial germination, reduced conidial yields, and decreased expression levels of some genes involved in conidiation. Furthermore, the *MrUbp14* mutant (Δ*MrUbp14*) exhibited decreased tolerance to cell wall-damaging stressors (Congo red and SDS) and heat stress. Importantly, the results of the bioassay demonstrated that the fungal virulence of the Δ*MrUbp14* strain was largely reduced in cuticle infection, but not in direct injection, which was accompanied by a significant decline in appressorium formation and cuticle penetration. Moreover, our results demonstrated that the disruption of *MrUbp14* resulted in significantly increased ubiquitination levels of total protein, suggesting that MrUbp14 acts as a deubiquitinating enzyme in *M. robertsii*. In summary, our phenotypic changes in the gene disruption mutants suggest that *MrUbp14* is important for conidiation, stress response, and fungal virulence in *M. robertsii*.

## Introduction

The ubiquitination of target substrates is accomplished through a series of steps catalyzed by three enzymes: E1 (ubiquitin (a highly conserved protein with 76 amino acid residues)-activating enzyme), E2 (ubiquitin-conjugating enzyme), and E3 (ubiquitin-protein ligases) (Srikanta and Cermakian, 2021). Protein ubiquitination is an important post-translational modification in eukaryotic cells that targets certain proteins for degradation by the ubiquitin-proteasome system (UPS), and thus has important roles in diverse biological processes involved in DNA repair, stress response, growth, and development (Brinkmann et al., 2015; Collins and Goldberg, 2017).

Ubiquitination isuitination is a reversible process because it is mediated by deubiquitinating enzymes (DUBs) (Suresh et al., 2020; Snyder and Silva, 2021). DUBs can function by removing ubiquitin from targeted proteins or by decreasing the length of polyubiquitin chains (Li and Ye, 2008). The balance between deubiquitination and ubiquitination has different effects on physiological protein abundance and activity; thus, dysregulation of DUBs will result in corresponding functional consequences (Suresh et al., 2020; Snyder and Silva, 2021). Moreover, because of the highly adaptive and reversible nature of ubiquitin signaling, DUBs are often involved in modulating protein function in response to developmental dynamics, environmental changes, or stress (Clague and Urbe, 2017). Therefore, similar to ubiquitination-associated enzymes, DUBs are important modifiers in the UPS and affect various biological processes (Callis, 2014; Cao and Xue, 2021).

It has been previously reported that DUBs are divided into five classes in yeast (according to structural and sequence similarity) (Suresh et al., 2020): the ubiquitin-specific proteases (UBPs/USPs); ubiquitin C-terminal hydrolases (UCHs); ovarian tumor proteases (OTUs); JAB1/MPN/MOV34s (JAMMs); and the recently discovered MINDY. In addition, USPs/UBPs, UCHs, OTUs, and MINDYs are cysteine proteases, whereas the JAMMs are metalloproteases, according to their action of catalysis (Clague et al., 2019). As a large family of DUBs, UBPs are involved in many physiological processes, such as enzymatic activities, ubiquitin homeostasis, and protein–protein interactions, through the regulation of the UPS (Collins and Goldberg, 2017; Suresh et al., 2020; Snyder and Silva, 2021). In *Saccharomyces cerevisiae*, UBPs modulate various developmental and stress responses (Kahana, 2001). For example, Ubp10 and Ubp15 are required for growth and stress response (Yoshikawa et al., 2011). In particular, disruption of *UBP14* results in defects in sporulation, which is accompanied by the accumulation of free ubiquitin (Amerik et al., 1997). These results suggest that Ubp14 maintains ubiquitin homeostasis in yeast (Amerik et al., 1997). Moreover, the deubiquitinating enzyme UBP5 is required for pathogenicity in the pathogenic fungi *Cryptococcus neoformans* and *Cryptococcus gattii* (Fang et al., 2012; Meng et al., 2016). Furthermore, it has been reported that 11 putative UBP genes and their biological roles were determined in the plant pathogenic fungi *Magnaporthe oryzae* (Cai et al., 2020; Wang et al., 2022). For instance, the disruption of *MoUBP14* leads to virulence attenuation and phenotypic defects, such as decreased conidiation and stress sensitivity (Wang et al., 2018). Additionally, the disruption of *MoUbp3, MoUbp4*, and *MoUbp8* leads to reduced penetration and invasive growth and thus reduced fungal virulence (Cai et al., 2020, 2022). However, it is not understood whether UBPs, especially Ubp14, are required for the growth, development, and virulence of fungal insect pathogens.

*Metarhizium robertsii* is an insect pathogenic fungus that is widely used for the biological control of diverse insect pests (Wang and Wang, 2017) and has become a model fungus for studying insect-fungal pathogen interactions in fungal insect pathogens (Guo et al., 2017). Previously, functional analysis of some ubiquitin-related genes has been performed in entomopathogenic fungi (Wang et al., 2019b; Wang D. Y. et al., 2021). In this study, we investigated the biological role of *MrUbp14* (MAA_07752) in *M. robertsii*.

## Materials and Methods

### Fungal Strains

Wild-type (WT) *M. robertsii* ARSEF 2575 and relative mutants were cultured on potato dextrose agar (PDA) plates at 25°C. To collect the conidia and prepare a conidial suspension (0.05% Tween-80), the fungus was cultured on PDA at 25°C for 10 days.

### Sequence Analysis

To identify the relationship between MrUbp14 and its orthologs, the corresponding amino acid sequences from different fungi were obtained from the National Center for Biotechnology Information (NCBI, http://www.ncbi.nlm.nih.gov/), and phylogenetic analysis was conducted using MEGA7 (Kumar et al., 2016). In addition, the conserved protein domains were investigated using SMART (http://smart.embl-heidelberg.de/).

### Gene Disruption

Primers used for gene deletion were designed according to a previous report (Wang et al., 2019a; Wang Z. X. et al., 2021) and are listed in Supplementary Table 1. The 5-flanking sequences (1,011 bp, with primer set MrUbp14-5F/MrUbp14-5R, enzyme digestion sites, *Bam*HI, are introduced) and 3′-flanking sequences (1,240 bp, with primer set MrUbp14-3F/MrUbp14-3R, enzyme digestion sites, *Xba*I, were introduced) of *MrUbp14* were amplified from genomic DNA using high-fidelity Taq DNA polymerase (KOD Plus Neo, Toyobo, Osaka, Japan) (Supplementary Figure 1A), and the resulting PCR products were cloned into the binary vector pDHt-SK-bar (conferring resistance to glufosinate-ammonium) to generate the *MrUbp14* replacement vector, p-bar-*MrUbp14*, which was used for fungal transformation to generate the deletion mutant Δ*MrUbp14* (Fang et al., 2006). Putative Δ*MrUbp14* mutants were screened by PCR and real-time PCR (RT-PCR) (Supplementary Figures 1B,C).

For gene complementation, the MrUbp14 gene together with its upstream and downstream regions was amplified with the primer MrUbp14Comp-5F/MrUbp14Comp-3R (as shown in Supplementary Table 1) and then cloned into the vector pDHt-SK-ben to generate the p-ben-MrUbp14 vector for fungal transformation (using Δ*MrUbp14* as background). Putative complementary mutants (Comp) were selected using PCR and RT-PCR (Supplementary Figures 1B,C).

### Growth, Development, and Stress Response Assays

For mycelial growth, conidial suspensions (1 μl) of relative strains were applied to PDA, SDAY, and 1/4 SDAY plates, and the size of the fungal colonies was measured after culturing for 14 days at 25°C. For conidial germination, conidial suspensions (10 μl) of the relative strains were applied to the center of the PDA plates. This was measured by microscopic counting conducted after every 2 h of incubation for 24 h, and the mean 50% germination time (GT_50_) was calculated by Kaplan–Meier analysis using SPSS software (SPSS, Inc., USA). In total, 300 conidia were collected per sample. For the conidiation assay, conidial suspensions (40 μl) were spread on PDA plates and incubated for 7 and 14 days at 25°C. The concentration of the conidial suspensions was measured using a hemocytometer according to our previous research (Wang et al., 2019a; Wang Z. X. et al., 2021).

For chemical stress tolerance, conidial suspensions (1 μl) of relative strains were applied to PDA plates alone (control) or PDA plates with the corresponding stressor Congo red (CR), sodium dodecyl sulfate (SDS) to destabilize cell wall integrity, menadione for oxidative stress, and NaCl for hyperosmotic stress. The size of fungal colonies was measured after culturing for 10 days at 25°C. Growth inhibition rates were determined as described in our previous study (Wang Z. X. et al., 2021).

For conidial tolerance to heat stress, conidial suspensions (1 ml) in 1.5 ml Eppendorf tubes were placed in a water bath at 42°C for different amounts of time. After incubation, 10 μl aliquots of the treated suspensions were added to PDA plates and incubated at 25°C. The rates of conidial germination were measured after culturing on PDA for 20 h. The IT_50_ (time for 50% inhibition of germination rate by heat treatment) for different strains was analyzed using GraphPad Prism software (Li et al., 2020; Wang Z. X. et al., 2021).

### Virulence Assays

For fungal virulence assays, immersion infection and direct injection were conducted according to our previous studies (Wang et al., 2019a; Wang Z. X. et al., 2021). For immersion infection, conidia were inoculated by dipping the *Galleria mellonella* larvae into a conidial suspension (1 × 10^7^ conidia ml^−1^) for 90 s. For direct injection, conidia were inoculated directly with a conidial suspension (5 μl of 5 × 10^5^ conidia ml^−1^) into the hemocoel of each larva. Mortality was measured every 24 h, and then the median lethal time (LT_50_) was calculated by Kaplan–Meier analysis using SPSS software (SPSS, Inc., USA).

For appressorium formation, conidial suspensions were applied to the wings of the cicada to generate appressorium, and the rates of appressorium formation were measured by examining 300 conidia per strain based on our previous research (Wang et al., 2019a). For cuticle penetration, conidial suspensions were applied to the center of an intact cicada wing (on a PDA plate) and then incubated for 48 h at 25°C. Subsequently, the cicada wings were removed, the PDA plate was cultured for 5 days at 25°C, and the size of the fungal colonies was measured and photographed according to our previous research (Wang Z. X. et al., 2021).

### Quantitative RT-PCR Analysis

To analyze the expression of conidiation-associated genes, quantitative analysis was performed according to previous reports (Wang et al., 2019a; Wang Z. X. et al., 2021). Total RNA was extracted with TRIzol Reagent (Invitrogen, Carlsbad, CA, USA) from fungal samples cultured on PDA plates for 2.5 days (initial conidiation stage) by inoculation of 100 μl conidial suspensions. The quantitative PCR (qPCR) (specific primers in Supplementary Table 2) was conducted using a CFX96^TM^ RT-PCR System (Bio-Rad, Hercules, CA, USA) with SYBR^®^ Premix Ex Taq™ II (TaKaRa, Dalian, China). The relative expression levels of the genes were investigated using the 2^−Δ*ΔCT*^ method (Livak and Schmittgen, 2001).

### Western Blot Analysis

For western blotting, the total proteins of each strain were extracted using a protein lysis buffer (Sangon, Shanghai, China) according to a previous report (Que et al., 2020). Total protein samples (15 μl) were separated on a 10% sodium dodecyl-sulfate polyacrylamide gel electrophoresis (SDS-PAGE) gel and transferred to a polyvinylidene fluoride (PVDF) membrane. Total ubiquitination levels were analyzed using an anti-ubiquitin antibody (PTM Biolabs Inc, PTM-1106), and β-tubulin was detected with an anti-beta tubulin antibody (Hangzhou HuaBio, M1305-2), which was used as an internal control.

## Results

### Sequence Analysis and Deletion of the *M. robertsii* Gene *MrUbp14*

**Figure 1:**
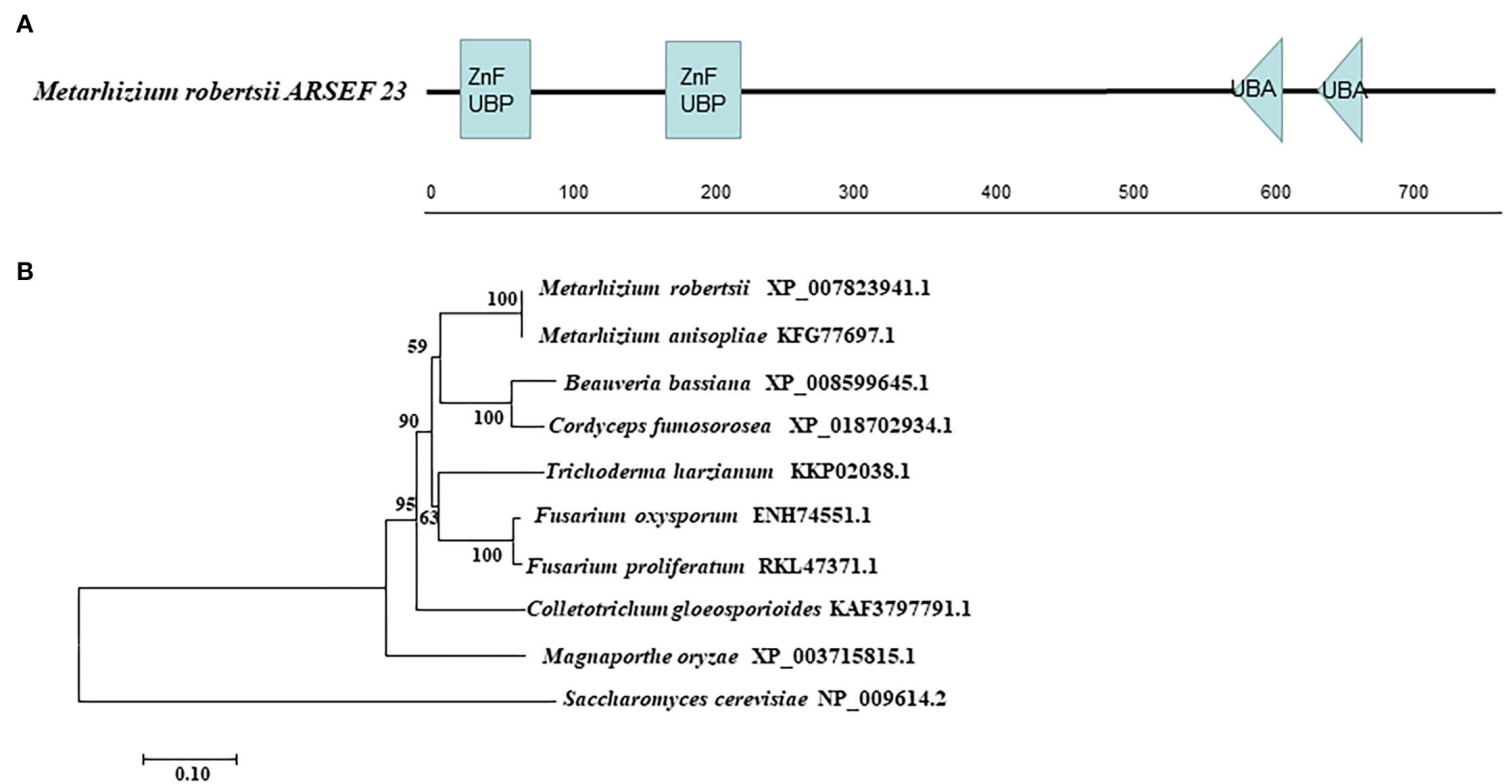
Sequence analysis of *MrUbp14*. **(A)** Conserved domain analysis of MrUbp14. ZnF_UBP, UBP-type Zinc finger domain. UBA, ubiquitin-associated domains. **(B)** Phylogenetic tree of MrUbp14 with their homologs. The phylogenetic analysis was obtained by the maximum likelihood method (using MEGA7 software based on default settings with 1,000 bootstrap replications). The corresponding accession numbers are followed by the names of fungal species. The bar indicates 0.1 distance units.

The enzyme gene *MrUbp14* (MAA_07752) was identified from the *M. robertsii* genome by BLASTp, using *S. cerevisiae* Ubp14 protein (NP_009614.2) as the query sequence. The coding sequence (2,343bp) of *MrUbp14* in the *M. robertsii* genome encodes a 780 amino acid protein (NCBI Accession Number: XP_007823941.1) with an isoelectric point of 5.11 and a molecular mass of 86.41 kDa. Domain analysis showed that MrUbp14 contains two ZnF_UBP domains (i.e., a UBP-type zinc finger domain) at the N-terminus and two ubiquitin-associated (UBA) domains at the C-terminus (Figure 1A). The phylogenetic tree of Ubp14 orthologs in fungi demonstrated that MrUbp14 has a closer evolutionary relationship with *Metarhizium anisopliae* (KFG77697.1), *Cordyceps fumosorosea* (XP_018702934.1), and *Beauveria bassiana* (XP_008599645.1), with positive amino acid identities of 99.87, 80.38, and 79.23%, respectively (Figure 1B).

To investigate the biological role of *MrUbp14* in *M. robertsii*, targeted gene deletion of *MrUbp14* was performed by homologous recombination (Supplementary Figure 1A). The Δ*MrUbp14* transformants were verified by PCR and RT-PCR analyses for *MrUbp14* (Supplementary Figures 1B,C), and the complemented strain with a genomic copy of *MrUbp14* was subsequently obtained based on the Δ*MrUbp14* background (Supplementary Figures 1B,C).

### *MrUbp14* Is Important for Conidiation in *M. robertsii*

**Figure 2:**
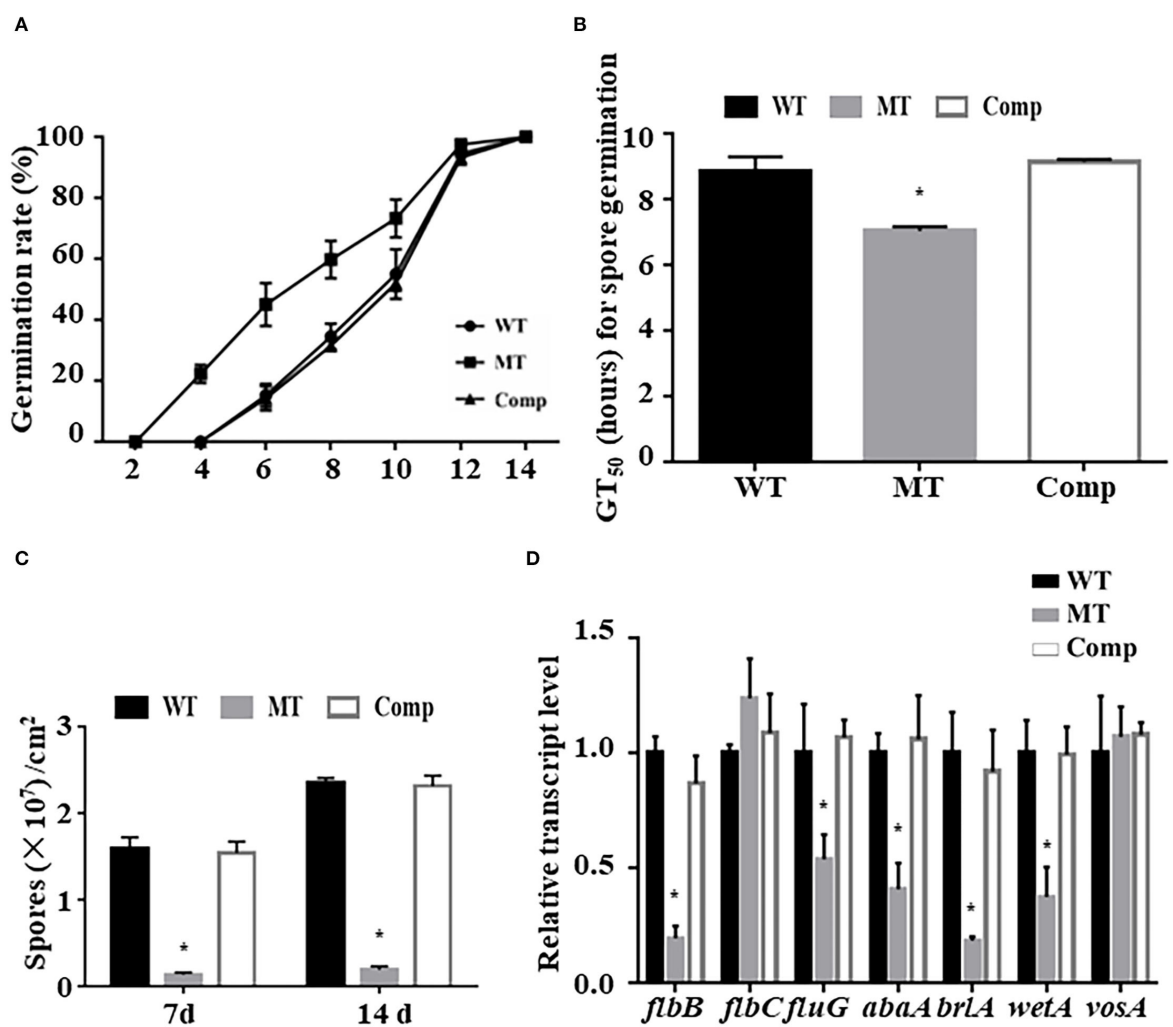
Impact of *MrUbp14* deletion on conidial germination and conidiation. **(A)** Conidial germination rate. The rates of conidial germination for relative strains were measured after inoculation at different times. **(B)** Half-time of germination (GT_50_) for relative strains incubated on potato dextrose agar (PDA) at 25°C for 24 h. **(C)** Conidial yields for relative strains after cultivation on PDA for different time points. **(D)** Expression levels of relative genes of different strains during initial conidiation stage. Error bars indicate standard deviation (SD) of three biological replicates. The asterisk (*) indicates *p* < 0.05.

To test the effect of *MrUbp14* deletion on conidiation-related events in *M. robertsii*, the effect of *MrUbp14* deletion on conidial germination was measured, and the results showed that the conidia of Δ*MrUbp14* displayed accelerated germination compared with the control strains (Figure 2A). For example, the mean 50% germination time (GT_50_) of Δ*MrUbp14* (7.03 ± 0.13 h) was markedly lower than that of the control strains (8.83 ± 0.46 h for WT and 9.14 ± 0.07 h for Comp strain) (Figure 2B). Second, conidial production of related strains showed that disruption of *MrUbp14* resulted in a substantial decline in conidial yields (Figure 2C). For instance, the conidial yields in 14-day-old cultures of the WT, Δ*MrUbp14*, and Comp strains were measured as (2.36 ± 0.05) × 10^7^, (0.19 ± 0.04) × 10^7^, and (2.31 ± 0.12) × 10^7^ conidia cm^−2^, respectively, suggesting a significant 91.95% decrease in the deletion of *MrUbp14*. In particular, some conidiation-associated genes in Δ*MrUbp14* were markedly repressed at the transcriptional level, such as *brlA, abaA, wetA, flbB*, and *fluG*, which are important for conidiation in filamentous fungi (Figure 2D).

In addition, to explore whether *MrUbp14* is required for vegetative growth, the colony morphology and size of different strains were observed on PDA, SDAY, and 1/4 SDAY (Supplementary Figure 2A). Our results showed that the phenotypes and growth diameter of the Δ*MrUbp14* colony were the same as those of the control strains on PDA, SDAY, and 1/4 SDAY (Supplementary Figure 2B).

### Deletion of *MrUbp14* Affects Stresses Tolerance

**Figure 3:**
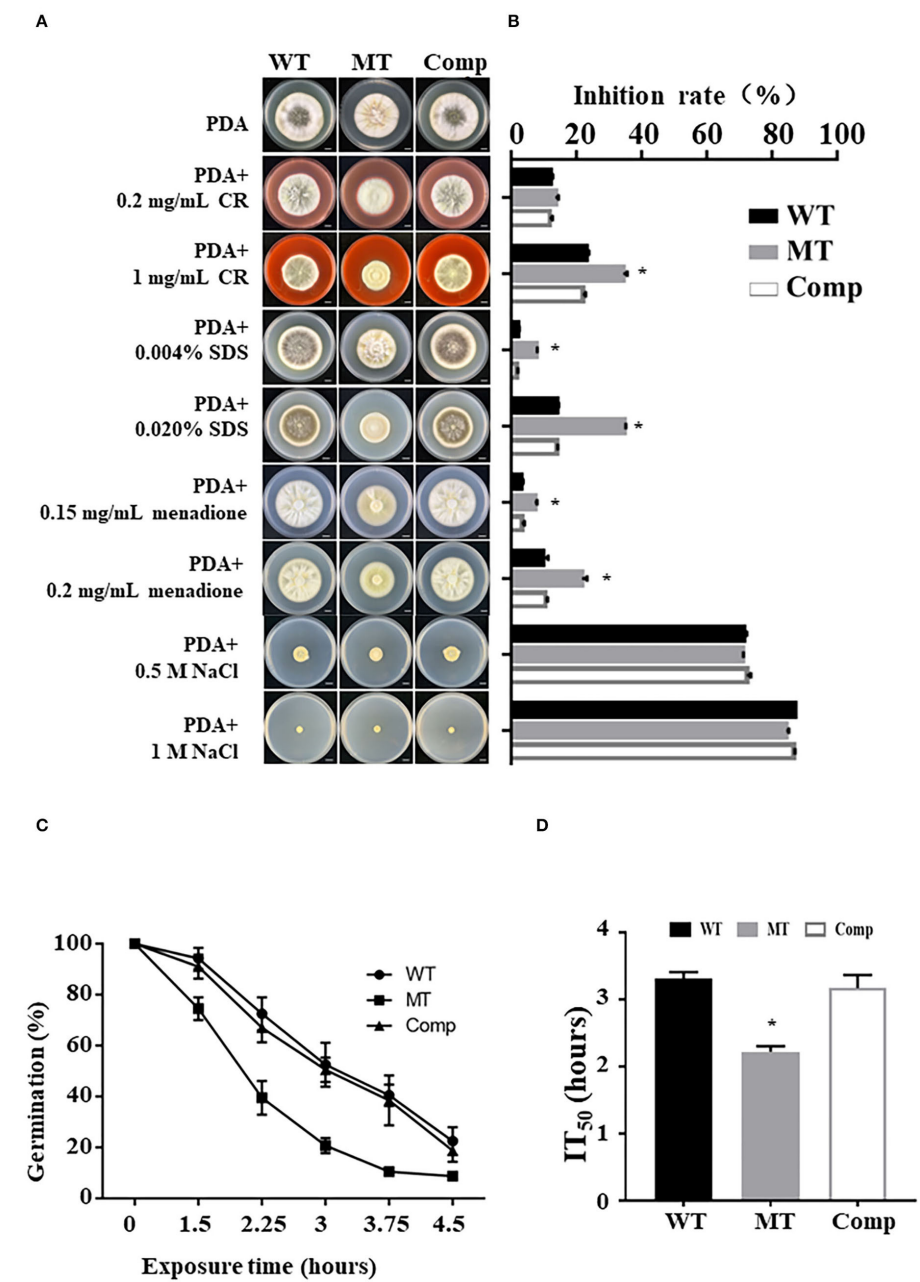
Effect of *MrUbp14* disruption on abiotic stress tolerance. **(A)** Phenotype of colony from relative strains incubated on PDA with chemical stressors. The images were obtained after cultivation at 25°C for 10 days. Scale: 1 cm. **(B)** The growth inhibition rates of different strains in the chemical stressor. Growth inhibition rate = (*D*_control_-*D*_treated_)/*D*_control_) × 100% (of which, *D* indicates the colony diameter of relative strains cultured on PDA at 25°C for 10 days). **(C)** The percentage of conidial germination for different strains were presented during heat stress. Conidia of relative strains were exposed to 45°C for different times, and then cultured on PDA at 25°C for 20 h to determine conidial germination. **(D)** The mean 50% inhibition time (IT_50_) of different strains under heat treatment. Error bars indicate SD of three biological replicates. The asterisk (*) indicates *p* < 0.05.

To explore the role of *MrUBP14* under chemical stress, a vegetative growth assay was conducted on PDA plates supplemented with diverse chemical stressors, such as CR, SDS, menadione, and NaCl (Figure 3A), and the relative growth inhibition was calculated by measuring colony diameters (Figure 3B). The results showed that the Δ*MrUBP14* strain exhibited increased sensitivity to cell wall integrity agents (CR and SDS) and oxidative stressors (menadione), whereas the chemical stressor NaCl did not affect the growth of Δ*MrUBP14* (Figures 3A,B).

Moreover, the effect of *MrUbp14* deletion on conidial survival under environmental stress was analyzed according to the germination rates of conidia over time, and our results demonstrated that conidial germination of Δ*MrUbp14* was largely decreased under heat stress (Figure 3C). Further analysis for the mean 50% inhibition time (IT_50_) of Δ*MrUbp14* was 2.22 ± 0.09 h, which is significantly reduced relative to control strains [WT (3.31 ± 0.10 h) or Comp (3.17 ± 0.20 h)] (Figure 3D). These results indicate that *MrUbp14* contributes to conidial thermotolerance in *M. robertsii*.

### *MrUbp14* Is Essential for Pathogenicity Through Cuticle Penetration

**Figure 4:**
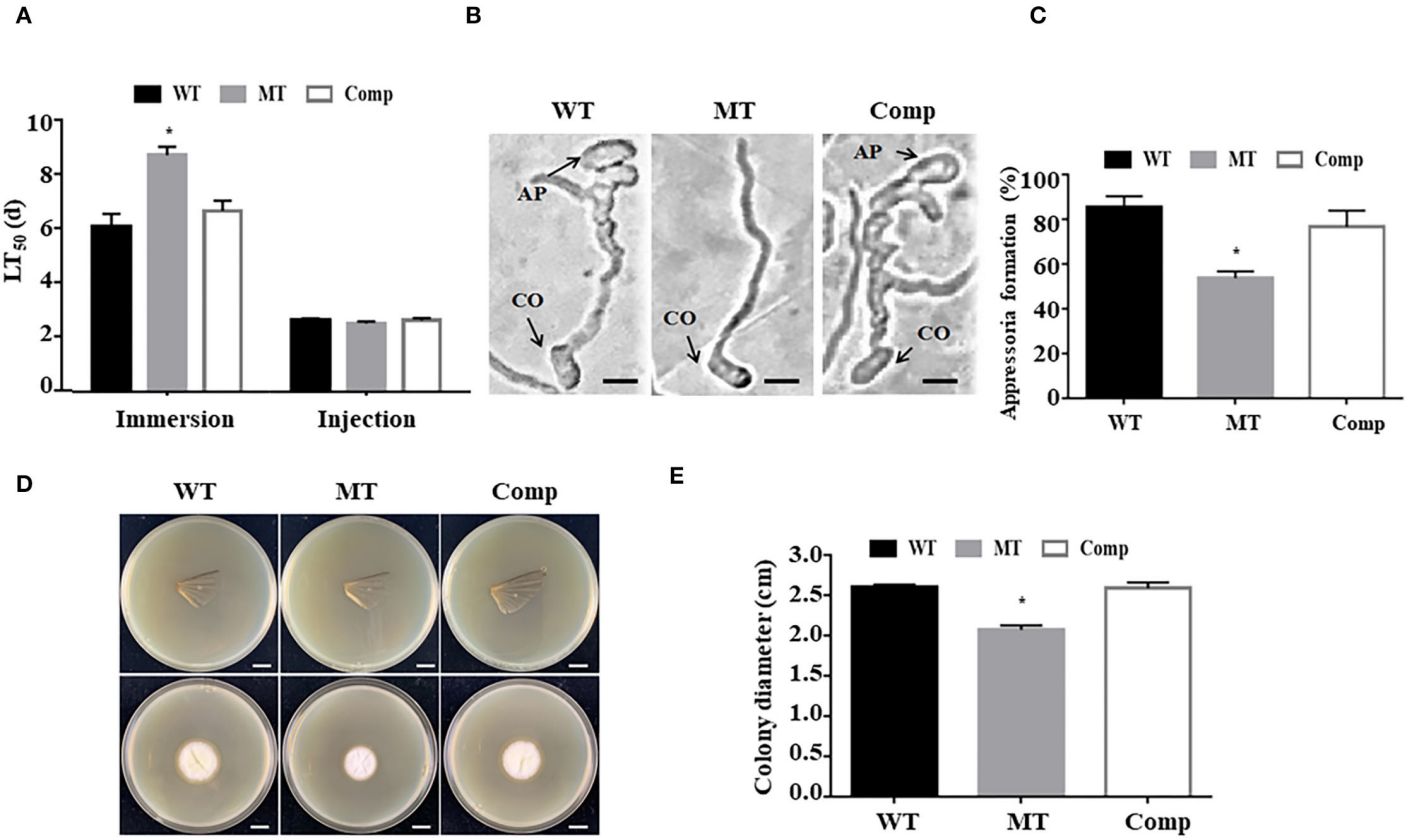
Effect of *MrUbp14* disruption on fungal virulence. **(A)** The median lethal time (LT_50_) (days) of different strains after topical inoculation and direct injection. **(B)** The images of appressorium formation generated on cicada wings. Scale: 10 μm. CO, conidium, AP, appressorium. **(C)** The percentage of appressorium formation generated on cicada wings. **(D)** The colony growth was measured after the removal of the cicada wings and incubation at 25°C for 5 days. Scale: 1 cm. **(E)** The colony diameters of relative strains cultured in the **(D)** section. Error bars indicate SD of three biological replicates. The asterisk (*) indicates *p* < 0.05.

To investigate the function of *MrUbp14* in fungal virulence, bioassays were conducted using conidial suspensions for direct injection and immersion infection. In immersion infection bioassays, the application of Δ*MrUbp14* led to a higher survival rate of treated larvae than the application of the WT and Comp strains. The LT_50_ of Δ*MrUbp14* was 8.71 ± 0.31 days, which was markedly longer than that of WT (6.07 ± 0.47 days) or Comp (6.65 ± 0.38 days) (Figure 4A). However, in the direct injection bioassays, it was approximately the same LT_50_ for Δ*MrUbp14* (2.51 ± 0.07 days) and WT (2.62 ± 0.07 days) or Comp (2.63 ± 0.09 days) (Figure 4A).

To explore the function of *MrUbp14* in appressorium formation, conidial suspensions were used on the surfaces of cicada wings to generate appressorium. Our results demonstrated that the induced rate of appressorium formation of Δ*MrUbp14* was significantly lower than that of the control strains (Figures 4B,C). Additionally, according to the cuticle penetration assay, the cuticle penetration ability of Δ*MrUbp14* was dramatically reduced (Figures 4D,E).

### Disruption of *MrUbp14* Results in Increased Total Ubiquitination Levels

**Figure 5:**
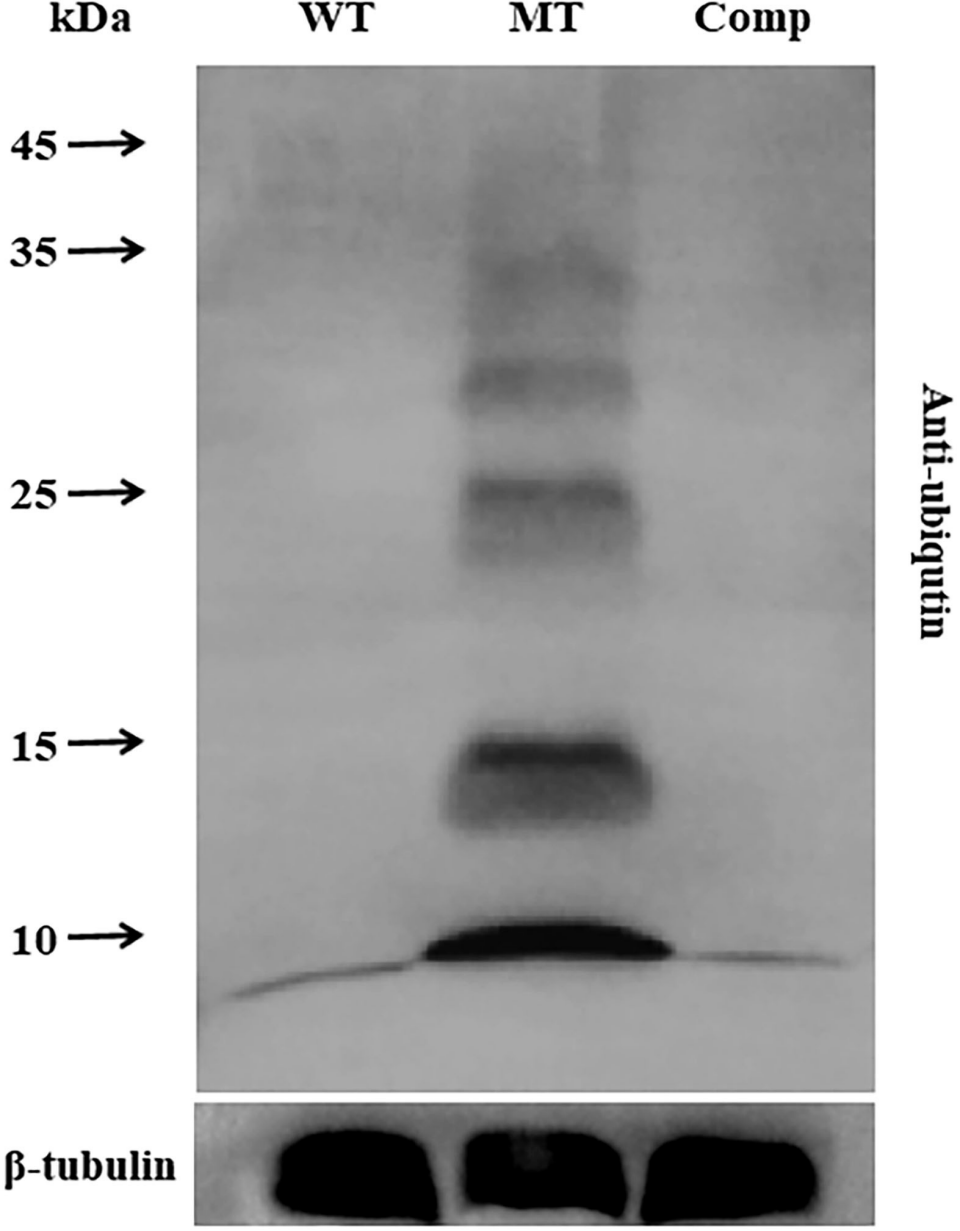
Effect of *MrUbp14* disruption on protein ubiquitination levels. Total proteins were obtained from the mycelia (cultured for 2 days on PDA) of each strain. Protein extracts were separated by 10% sodium dodecyl-sulfate polyacrylamide gel electrophoresis (SDS-PAGE) and then probed with anti-ubiquitin antibodies (PTM bio) to determine total ubiquitin levels. The ubiquitination levels of total proteins from different strains were compared using western blot analysis. An anti-tubulin antibody was used as an internal control. Disruption of *MrUbp14* leads to a large increase in the ubiquitination levels of total protein.

To analyze the role of MrUbp14 as a deubiquitinase, the ubiquitination levels of total protein in the different strains were investigated. Total proteins were obtained from mycelia incubated on PDA plates for 2 days, and ubiquitination levels were measured by western blotting. Our results showed that the ubiquitination levels of total protein from Δ*MrUbp14* were largely upregulated, and mono-ubiquitin was also dramatically increased compared with WT and Comp strains (Figure 5). These results indicate that disruption of *MrUbp14* led to a significant increase in protein ubiquitination levels.

## Discussion

Protein ubiquitination is important for modulating protein abundance in diverse eukaryotic cellular processes through a series of steps that are catalyzed by ubiquitin-related enzymes (Finley et al., 2012). DUBs can maintain ubiquitin homeostasis, which modulates the removal and processing of ubiquitin (Kim et al., 2003; Suresh et al., 2020; Snyder and Silva, 2021). Recent studies have demonstrated that the ubiquitin system and ubiquitin-related genes play important roles in the development and virulence of fungal pathogens (McCafferty and Talbot, 1998; Oh et al., 2012; Wang et al., 2019b, 2020; Wang D. Y. et al., 2021). However, little is known about the deubiquitination process. In this study, we identified a yeast Ubp14 ortholog, MrUbp14, in *M. robertsii*. Similar to yeast Ubp14 (NP_009614.2), MrUbp14 contains a conserved ZnF_UBP domain at the N-terminus and a UBA domain at the C-terminus (Figure 1A). More importantly, the significance of the deubiquitinase gene *MrUbp14* was investigated in *M. robertsii*, and the results demonstrated that *MrUbp14* contributes to deubiquitination, conidiation, stress response, and fungal virulence in *M. robertsii*.

As the largest group of DUBs, UBPs are involved in growth and development. Recent reports have revealed that several UBPs are critical for many developmental processes in yeast (Nanyan et al., 2020; Suresh et al., 2020) and *M. oryzae* (Cai et al., 2020; Wang et al., 2022). For instance, Ubp15 was found to be required for growth and development in yeast (Yoshikawa et al., 2011), and the loss of yeast UBP14 resulted in defects in sporulation (Amerik et al., 1997). In *M. oryzae*, most UBP gene deletion strains (such as *Ubp3, Ubp4*, and *Ubp14*) displayed decreased colony growth or conidial production (Cai et al., 2020). In this study, our data showed that the loss of *MrUbp14* resulted in reduced conidial yields, which was also accompanied by decreased expression levels of some conidiation-requiring genes, such as *wetA, brlA, abaA, flbB*, and *fluG*. In addition, the Δ*MrUbp14* mutant exhibited accelerated conidial germination. These results suggest that MrUbp14 affects conidiation in *M. robertsii*.

Ubiquitin-specific proteases are also involved in virulence in several pathogenic fungi (Fang et al., 2012; Meng et al., 2016; Cai et al., 2020; Wang et al., 2022). For example, in the pathogenic fungi *C. neoformans* and *C. gattii*, disruption of the deubiquitinating enzyme UBP5 results in a severe decrease in virulence (Fang et al., 2012; Meng et al., 2016). In addition, it has recently been demonstrated that most members of the UBP genes (such as *Ubp3, Ubp4*, and *Ubp14*) play critical roles in the pathogenicity of *M. oryzae* (Cai et al., 2020; Wang et al., 2022). In this study, we found that the fungal virulence of the Δ*MrUbp14* strain was largely reduced by cuticle infection but not by direct injection, which is also consistent with our recent report on the deletion of *MrPEX33* (Wang Z. X. et al., 2021). According to a previous report on fungal insect pathogens (Gao et al., 2011; Wang and Wang, 2017), cuticle infection is involved in many processes, such as enzymatic hydrolysis and mechanical pressure, whereas direct injection is usually involved in the host immune response. Therefore, because of the difference in virulence between the immersion and injection assays, we speculate that the deletion of *MrUbp14* has an influence on cuticle penetration or appressorium formation, but there is no effect on the immune response of the host. It has been previously demonstrated that a significant decline in the appressorium formation was displayed, reducing the ability of cuticle penetration (Gao et al., 2016; Du et al., 2018; Wen et al., 2020). In this study, our data showed that MrUbp14 gene disruption strains exhibited reduced appressorium formation and cuticle penetration ability, which is consistent with previous studies (Gao et al., 2016; Du et al., 2018; Wen et al., 2020). In addition, previous reports have shown that the gene deletion of Ubp leads to a defect in cell wall integrity, which contributes to a loss of appressorial turgor pressure and cuticle penetration ability (Wang et al., 2018; Que et al., 2020). Indeed, the Δ*MrUbp14* mutant showed increased sensitivity to cell wall integrity agents (CR and SDS), suggesting an impairment in cell wall integrity. Based on these results, we reasoned that the loss of *MrUbp14* affects the deubiquitination process, which leads to a decrease in fungal virulence *via* cuticle penetration and impairment in cell wall integrity.

In conclusion, MrUbp14 serves as a deubiquitinase and plays an important role in conidiation, the stress response, and fungal virulence in *M. robertsii*. These findings will promote an understanding of the role of the deubiquitination process in entomopathogenic fungi.

## Data Availability Statement

The original contributions presented in the study are included in the article/Supplementary Material, further inquiries can be directed to the corresponding author/s.

## Author Contributions

ZW contributed to the conception and design of the study. HuC, HL, and HaC performed experiments and data analysis. ZW and HL prepared the manuscript. BH supervised and helped in the revision of the manuscript. All authors contributed to the article and approved the submitted version.

## Funding

This research was jointly supported by the Project for Excellent Young Talents in Universities of Anhui Province (Grant No. gxyqZD2020007), the National Natural Science Foundation of China (Grant Nos. 31972332, 32172473, and 31572060), and the Natural Science Foundation of Anhui Province (2108085MC101).

## Conflict of Interest

The authors declare that the research was conducted in the absence of any commercial or financial relationships that could be construed as a potential conflict of interest.

## Publisher's Note

All claims expressed in this article are solely those of the authors and do not necessarily represent those of their affiliated organizations, or those of the publisher, the editors and the reviewers. Any product that may be evaluated in this article, or claim that may be made by its manufacturer, is not guaranteed or endorsed by the publisher.

## References

[B1] Amerik A. Swaminathan S. Krantz B. A. Wilkinson K. D. Hochstrasser M. (1997). *In vivo* disassembly of free polyubiquitin chains by yeast Ubp14 modulates rates of protein degradation by the proteasome. EMBO J. 16, 4826–4838. 10.1093/emboj/16.16.4826 9305625 PMC1170118

[B2] Brinkmann K. Schell M. Hoppe T. Kashkar H. (2015). Regulation of the DNA damage response by ubiquitin conjugation. Front. Genet. 6, 98. 10.3389/fgene.2015.00098 25806049 PMC4354423

[B3] Cai X. Wang Z. Hou Y. X. Liu C. Y. Hendy A. Xing J. J.. (2020). Systematic characterization of the ubiquitin-specific proteases in *Magnaporthe oryzae*. Phytopathol. Res. 2, 8. 10.1186/s42483-020-00050-1

[B4] Cai X. Xiang S. K. He W. H. Tang M. X. Zhang S. M. Chen D.. (2022). Deubiquitinase Ubp3 regulates ribophagy and deubiquitinates Smo1 for appressorium-mediated infection by *Magnaporthe oryzae*. Mol. Plant Pathol. 10.1111/mpp.13196 35220670 PMC9104258

[B5] Callis J. (2014). The ubiquitination machinery of the ubiquitin system. Arabidopsis Book 12, e0174. 10.1199/tab.0174 25320573 PMC4196676

[B6] Cao C. J. Xue C. Y. (2021). More than just cleaning: ubiquitin-mediated proteolysis in fungal pathogenesis. Front. Cell. Infect. Microbiol. 11, 774613. 10.3389/fcimb.2021.774613 34858882 PMC8631298

[B7] Clague M. J. Urbe S. (2017). Integration of cellular ubiquitin and membrane traffic systems: focus on deubiquitylases. FEBS J. 284, 1753–1766. 10.1111/febs.14007 28064438 PMC5484354

[B8] Clague M. J. Urbe S. Komander D. (2019). Breaking the chains: deubiquitylating enzyme specificity begets function. Nat. Rev. Mol. Cell Biol. 20, 338–352. 10.1038/s41580-019-0099-1 30733604

[B9] Collins G. A. Goldberg A. L. (2017). The logic of the 26S proteasome. Cell 169, 792–806. 10.1016/j.cell.2017.04.023 28525752 PMC5609836

[B10] Du Y. R. Jin K. Xia Y. X. (2018). Involvement of *MaSom1*, a downstream transcriptional factor of cAMP/PKA pathway, in conidial yield, stress tolerances, and virulence in *Metarhizium acridum*. Appl. Microbiol. Biotechnol. 102, 5611–5623. 10.1007/s00253-018-9020-7 29713793

[B11] Fang W. Price M. S. Toffaletti D. L. Tenor J. Betancourt-Quiroz M. Price J. L.. (2012). Pleiotropic effects of deubiquitinating enzyme Ubp5 on growth and pathogenesis of *Cryptococcus neoformans*. PLoS ONE 7, e0038326. 10.1371/journal.pone.0038326 22719877 PMC3375289

[B12] Fang W. G. Pei Y. Bidochka M. J. (2006). Transformation of *Metarhizium anisopliae* mediated by *Agrobacterium tumefaciens*. Can. J. Microbiol. 52, 623–626. 10.1139/w06-014 16917517

[B13] Finley D. Ulrich H. D. Sommer T. Kaiser P. (2012). The ubiquitin-proteasome system of *Saccharomyces cerevisiae*. Genetics 192, 319–360. 10.1534/genetics.112.140467 23028185 PMC3454868

[B14] Gao Q. Lu Y. Z. Yao H. Y. Xu Y. J. Huang W. Wang C. S. (2016). Phospholipid homeostasis maintains cell polarity, development and virulence in *Metarhizium robertsii*. Environ. Microbiol. 18, 3976–3990. 10.1111/1462-2920.13408 27312218

[B15] Gao Q. A. Jin K. Ying S. H. Zhang Y. J. Xiao G. H. Shang Y. F.. (2011). Genome sequencing and comparative transcriptomics of the model entomopathogenic fungi *Metarhizium anisopliae* and M. acridum. PLoS Genet. 7, e1001264. 10.1371/journal.pgen.1001264 21253567 PMC3017113

[B16] Guo N. Qian Y. Zhang Q. Q. Chen X. X. Zeng G. H. Zhang X.. (2017). Alternative transcription start site selection in Mr-OPY2 controls lifestyle transitions in the fungus *Metarhizium robertsii*. Nat. Commun. 8, 1565. 10.1038/s41467-017-01756-1 29146899 PMC5691130

[B17] Kahana A. (2001). The deubiquitinating enzyme Dot4p is involved in regulating nutrient uptake. Biochem. Biophys. Res. Commun. 282, 916–920. 10.1006/bbrc.2001.4669 11352638

[B18] Kim J. H. Park K. C. Chung S. S. Bang O. Chung C. H. (2003). Deubiquitinating enzymes as cellular regulators. J. Biochem. 134, 9–18. 10.1093/jb/mvg107 12944365

[B19] Kumar S. Stecher G. Tamura K. (2016). MEGA7: molecular evolutionary genetics analysis version 7.0 for bigger datasets. Mol. Biol. Evol. 33, 1870–1874. 10.1093/molbev/msw054 27004904 PMC8210823

[B20] Li J. Guo M. Cao Y. Q. Xia Y. X. (2020). Disruption of a C69-family cysteine dipeptidase gene enhances heat shock and UV-B tolerances in *Metarhizium acridum*. Front. Microbiol. 11, 849. 10.3389/fmicb.2020.00849 32431687 PMC7214794

[B21] Li W. Ye Y. (2008). Polyubiquitin chains: functions, structures, and mechanisms. Cell. Mol. Life Sci. 65, 2397–2406. 10.1007/s00018-008-8090-6 18438605 PMC2700825

[B22] Livak K. J. Schmittgen T. D. (2001). Analysis of relative gene expression data using real-time quantitative PCR and the 2 ^−ΔΔCT^ method. Methods 25, 402–408. 10.1006/meth.2001.1262 11846609

[B23] McCafferty H. R. Talbot N. J. (1998). Identification of three ubiquitin genes of the rice blast fungus *Magnaporthe grisea*, one of which is highly expressed during initial stages of plant colonisation. Curr. Genet. 33, 352–361. 10.1007/s002940050347 9618586

[B24] Meng Y. F. Zhang C. Yi J. Zhou Z. J. Fa Z. Z. Zhao J. Y.. (2016). Deubiquitinase Ubp5 is required for the growth and pathogenicity of *Cryptococcus gattii*. PLoS ONE 11:e0153219. 10.1371/journal.pone.0153219 27049762 PMC4822882

[B25] Nanyan N. Watanabe D. Sugimoto Y. Takagi H. (2020). Effect of the deubiquitination enzyme gene UBP6 on the stress-responsive transcription factor Msn2-mediated control of the amino acid permease Gnp1 in yeast. J. Biosci. Bioeng. 129, 423–427. 10.1016/j.jbiosc.2019.10.002 31640922

[B26] Oh Y. Franck W. Gokce E. Muddiman D. C. Dean R. A. (2012). Polyubiquitin is required for growth, development, and pathogenicity in the rice blast fungus *Magnaporthe oryzae*. Phytopathology 102, 87–87. 10.1371/journal.pone.0042868 22900059 PMC3416782

[B27] Que Y. W. Xu Z. Wang C. Y. Lv W. Y. Yue X. F. Xu L.. (2020). The putative deubiquitinating enzyme MoUbp4 is required for infection-related morphogenesis and pathogenicity in the rice blast fungus *Magnaporthe oryzae*. Curr. Genet. 66, 561–576. 10.1007/s00294-019-01049-8 31872271

[B28] Snyder N. A. Silva G. M. (2021). Deubiquitinating enzymes (DUBs): Regulation, homeostasis, and oxidative stress response. J. Biol. Chem. 297, 101077. 10.1016/j.jbc.2021.101077 34391779 PMC8424594

[B29] Srikanta S. B. Cermakian N. (2021). To Ub or not to Ub: regulation of circadian clocks by ubiquitination and deubiquitination. J. Neurochem. 157, 11–30. 10.1111/jnc.15132 32717140

[B30] Suresh H. G. Pascoe N. Andrews B. (2020). The structure and function of deubiquitinases: lessons from budding yeast. Open Biol. 10, 200279. 10.1098/rsob.200279 33081638 PMC7653365

[B31] Wang C. S. Wang S. B. (2017). Insect pathogenic fungi: genomics, molecular interactions, and genetic improvements. Annu. Rev. Entomol. 62, 73–90. 10.1146/annurev-ento-031616-035509 27860524

[B32] Wang D. Y. Mou Y. N. Du X. Guan Y. Feng M. G. (2021). Ubr1-mediated ubiquitylation orchestrates asexual development, polar growth, and virulence-related cellular events in *Beauveria bassiana*. Appl. Microbiol. Biotechnol. 105, 2747–2758. 10.1007/s00253-021-11222-0 33686455

[B33] Wang D. Y. Ren K. Tong S. M. Ying S. H. Feng M. G. (2020). Pleiotropic effects of Ubi4, a polyubiquitin precursor required for ubiquitin accumulation, conidiation and pathogenicity of a fungal insect pathogen. Environ. Microbiol. 22, 2564–2580. 10.1111/1462-2920.14940 32056334

[B34] Wang Y. Yang N. Zheng Y. N. Yue J. L. Bhadauria V. Peng Y. L.. (2022). Ubiquitination in the rice blast fungus *Magnaporthe oryzae*: from development and pathogenicity to stress responses. Phytopathol. Res. 4, 1. 10.1186/s42483-021-00106-w29633464

[B35] Wang Z. Zhang H. Liu C. Y. Xing J. J. Chen X. L. (2018). A Deubiquitinating Enzyme Ubp14 Is Required for Development, Stress Response, Nutrient Utilization, and Pathogenesis of *Magnaporthe oryzae*. Frontiers in Microbiology 9. 10.3389/fmicb.2018.00769 29720973 PMC5915541

[B36] Wang Z. X. Feng J. Y. Jiang Y. Y. Xu X. Z. Xu L. Y. Zhou Q.. (2021). MrPEX33 is involved in infection-related morphogenesis and pathogenicity of *Metarhizium robertsii*. Appl. Microbiol. Biotechnol. 105, 1079–1090. 10.1007/s00253-020-11071-3 33443633

[B37] Wang Z. X. Jiang Y. Y. Li Y. D. Feng J. Y. Huang B. (2019a). MrArk1, an actin-regulating kinase gene, is required for endocytosis and involved in sustaining conidiation capacity and virulence in *Metarhizium robertsii*. Appl. Microbiol. Biotechnol. 103, 4859–4868. 10.1007/s00253-019-09836-6 31025075

[B38] Wang Z. X. Zhu H. Cheng Y. R. Jiang Y. Y. Li Y. D. Huang B. (2019b). The polyubiquitin gene *MrUBI4* is required for conidiation, conidial germination, and stress tolerance in the filamentous fungus *Metarhizium robertsii*. Genes 10, 412. 10.3390/genes10060412 31146457 PMC6627135

[B39] Wen Z. Q. Tian H. T. Xia Y. X. Jin K. (2020). MaPmt1, a protein O-mannosyltransferase, contributes to virulence through governing the appressorium turgor pressure in *Metarhizium acridum*. Fungal Genet. Biol. 145, 103480. 10.1016/j.fgb.2020.103480 33130254

[B40] Yoshikawa K. Tanaka T. Ida Y. Furusawa C. Hirasawa T. Shimizu H. (2011). Comprehensive phenotypic analysis of single-gene deletion and overexpression strains of *Saccharomyces cerevisiae*. Yeast 28, 349–361. 10.1002/yea.1843 21341307

