# Supplementary material for: The Deubiquitinating Enzyme MrUbp14 Is Involved in Conidiation, Stress Response, and Pathogenicity in Metarhizium robertsii
Source: Front Fungal Biol. 2022 May 16;3:896466. doi: 10.3389/ffunb.2022.896466 (PMC10512391; doi:10.3389/ffunb.2022.896466)
Supplement: Supplementary file 1 [file Data_Sheet_1.PDF]

SUPPLEMENTARY MATERIAL For

The deubiquitinating enzyme MrUbp14 is involved in conidiation, stress response and pathogenicity in *Metarhizium robertsii*

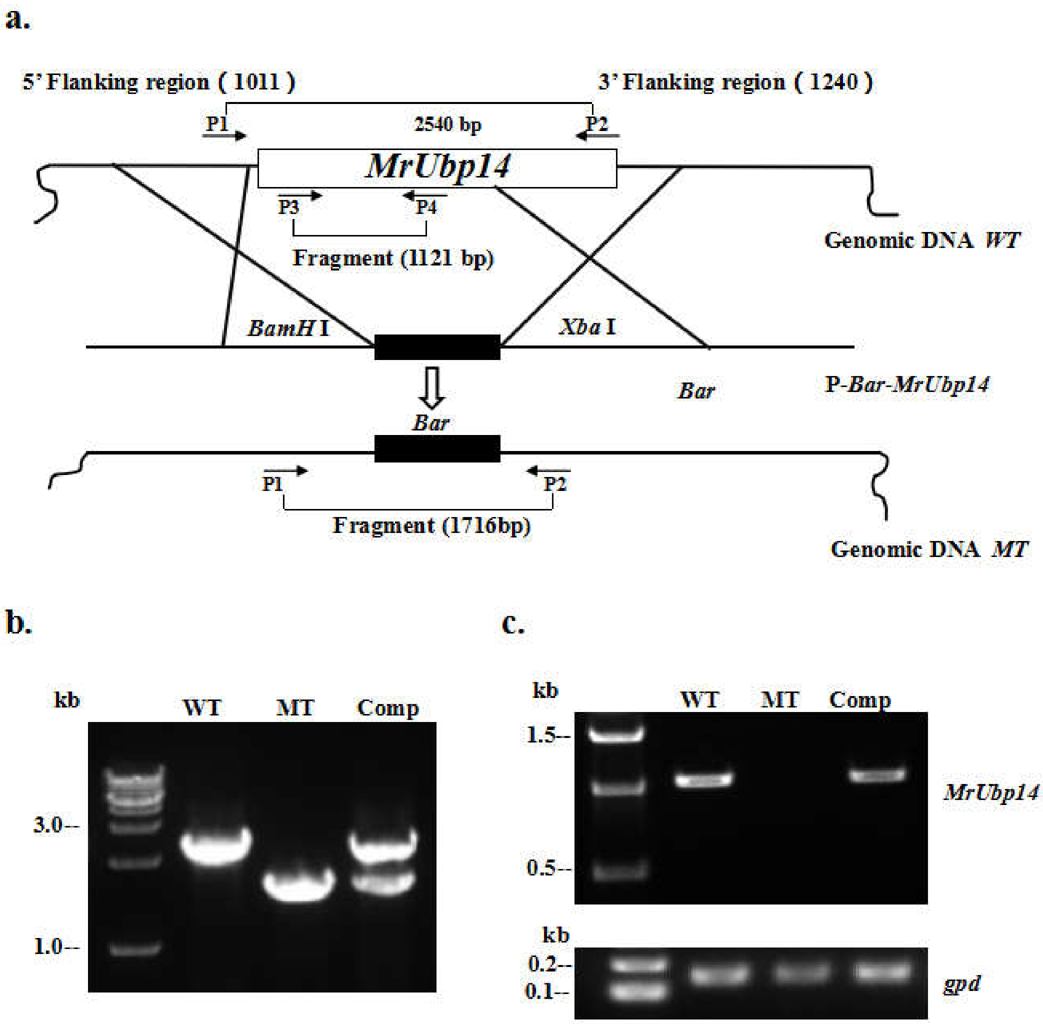

FIGURE S1 Gene deletion and complementation of *MrUbp14* in *M. robertsii*.

(A) Schematic diagram of *MrUbp14* disruption by homologous recombination approach. The part fragments of *MrUbp14* were replaced with 944-base pair of the *bar* cassette through homologous recombination between the overlapping regions of the 5′ and 3′ fragments (1011 and 1240 bp) in *MrUbp14* and pbar-*MrUbp14* (recombinant vector), respectively. The corresponding locations of the primers are indicated by arrows.

(B) PCR confirmation. Genomic DNAs extracted from different strains were used as templates for PCR. Transformants were verified by PCR analysis using primers sets P1/P2 (Table S1). The fragment (2540-bp) of *MrUbp14* was amplified with the primer sets P1/P2 from WT and Comp but not from Δ*MrUbp14*. The fragments (1716-bp) were amplified with the primer sets P1/P2 from Δ*MrUbp14* and Comp, after the part fragments of *MrUbp14* were replaced by 944-bp of the *bar* cassette. WT (wild-type strain), MT (Δ*MrUbp14* strain), Comp (complemented strain).

(C) Reverse transcription (RT)-PCR verification of gene expression of *MrUbp14* in WT, Δ*MrUbp14*, and Comp strains. Using cDNA as a template, the fragments (1121-bp) of *MrUbp14* was amplified with the primer sets P3/P4 (Table S1) from WT and Comp but not from Δ*MrUbp14*, which suggested that transcripts of *MrUbp14* were completely undetectable in the gene deletion mutant and were detected in the WT and Comp strains. The expression level of glyceraldehyde 3-phosphate dehydrogenase (*gpd*, MAA_07675) was used as an internal control. WT (wild-type strain), MT (Δ*MrUbp14* strain), Comp (complemented strain).

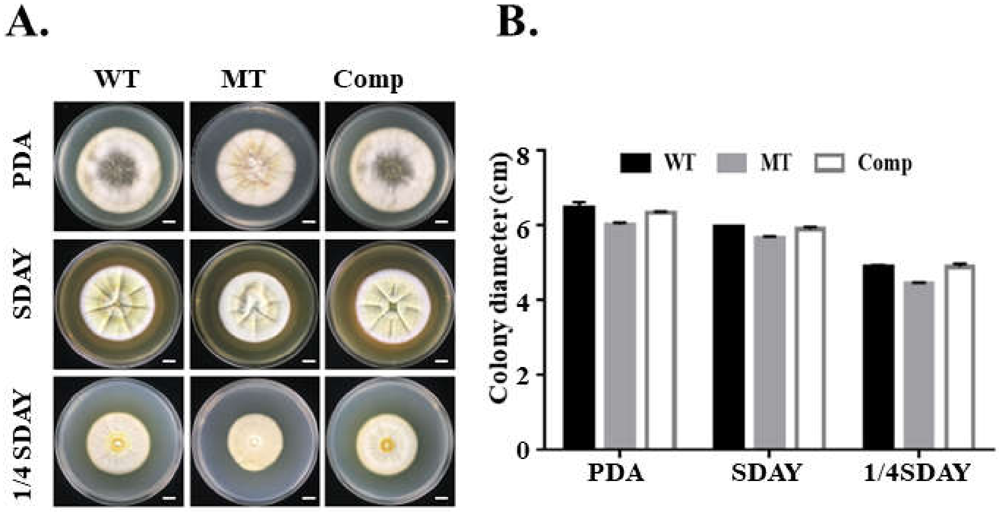

FIGURE S2 Effect of *MrUbp14* deletion on vegetative growth.

(A) Colony phenotyping of relative strains on different media after cultured at 25°C for 14 days. Scale: 1 cm.

(B) Colony diameters of relative strains on different media after cultured at 25°C for 14 days.

Table S1 Primers used in this study

Gene	Primer Name	Sequence (5'- 3')	Notes
Ubp14	*Ubp14*-5F	ATTCCTGCAGCCCGGGTAACAGTCAGGAGGGA, *Bam*HI	For construction of gene disruption vector
	Ubp14-5R	ATCATCTTCTGTCGACTCCTTAGCCTTCTTG, *Bam*HI	
	Ubp14-3F	GATCTGATGAACTAGTCCCGAGATTCTGGTTGTGA , *Xba*I	
	Ubp14-3R	CCGCGGTGGCGGCCGCCAGTAAACCGTCCCGCA, *Xba*I	
	Ubp14-F (P1)	CGACCTGCGCTCTAAC	PCR identification
	Ubp14-R (P2)	CGGCACCCTCTTCTTC	
	Ubp14-F (P3)	ATTAACCCCTCCGACT	RT-PCR analysis
	Ubp14-R (P4)	ACCGTCTCCCATCTTT	
	*Ubp14*Comp-5F	AAGCTTCGCACTAGTTCTAGCCCTCGTAACTCGC, *Xba*I	For gene complementation
	*Ubp14*Comp-3R	CGCGGTGGCGGCCGCTCTAGATGTAGATACCCTCGTCAGC , *Xba*I	
gpd	*gpd*-F	GACTGCCCGCATTGAGAAG	RT-PCR analysis
	gpd-R	AGATGGAGGAGTTGGTGTTG	

Table S2 Primers used for qRT-PCR analysis of conidiation-related genes

Gene	Accession number	Annotation	Sequence of primer sets (5'- 3')
flbB	MAA_00196	BZIP-type transcription factor	TCCACGCTGCTTGATT / CCTCACTTTGCGACCC
flbC	MAA_03655	Conidiophore development protein	AACGATGGGCTGAGATTG / GGTGATTGAGTTTCGGATG
fluG	MAA_00122	protein fluG	TGCGGGTTGAATACGG / CTCCACCTCTTTCTCCTTGA
abaA	MAA_00694	Conidiation transcription factor AbaA	AAACCACTATTCCTGCTCC / AGCCTGCCTGTTACGATA
brlA	MAA_10599	C_2H_2 conidiation transcription factor BrlA	CAACAGCAGGAATCGC / GCTTATCGGCTGACTTTG
wetA	MAA_02845	Conidial maturation factor WetA	CGACGAAATAGGAAAGCA / TGAAGTGGAGGAGATACGG
vosA	MAA_05862	Protein VosA	ACCGAAATCGTGAGTG / GTTGCCCTTCTTGATG
